# Nanophthalmos: A Review of the Clinical Spectrum and Genetics

**DOI:** 10.1155/2018/2735465

**Published:** 2018-05-09

**Authors:** Pedro C. Carricondo, Thais Andrade, Lev Prasov, Bernadete M. Ayres, Sayoko E. Moroi

**Affiliations:** ^1^Department of Ophthalmology, Hospital das Clínicas HCFMUSP, Faculdade de Medicina, Universidade de São Paulo, São Paulo, SP, Brazil; ^2^Department of Ophthalmology and Visual Sciences, Kellogg Eye Center, University of Michigan, 1000 Wall St., Ann Arbor, MI 48105, USA

## Abstract

Nanophthalmos is a clinical spectrum of disorders with a phenotypically small but structurally normal eye. These disorders present significant clinical challenges to ophthalmologists due to a high rate of secondary angle-closure glaucoma, spontaneous choroidal effusions, and perioperative complications with cataract and retinal surgeries. Nanophthalmos may present as a sporadic or familial disorder, with autosomal-dominant or recessive inheritance. To date, five genes (i.e., *MFRP*, *TMEM98*, *PRSS56*, *BEST1*, and *CRB1*) and two loci have been implicated in familial forms of nanophthalmos. Here, we review the definition of nanophthalmos, the clinical and pathogenic features of the condition, and the genetics of this disorder.

## 1. Introduction

The clinical spectrum of the small eye phenotype comprises conditions in which there is a global ocular reduction in size (e.g., microphthalmos and nanophthalmos) or shortening of either the anterior or posterior segments of the eye (e.g., relative anterior and posterior microphthalmos, resp.) (Table 1) [1–3]. The axial length and anterior chamber structures present a continuum of sizes (Table 2), where microphthalmos and nanophthalmos comprise the smallest or shortest eyes. Nanophthalmos derives from Greek “dwarf eye.” In this ocular condition, the anterior and posterior segments have no other congenital malformations, but are both reduced in size, with secondary thickening of choroid and sclera.

The management of the small eye phenotype represents a major challenge for all ophthalmologists, from cataract surgeons to glaucoma and retina specialists. Small eyes may be associated with ophthalmic or systemic comorbidities. These eyes represent significant surgical challenges with a very high rate of intraoperative complications [4] and require a surgical approach that involves precision and care. Recognizing and correctly diagnosing the diverse presentations of this condition is of great importance for appropriate clinical and surgical management. Understanding the genetic mechanisms involved in the pathogenesis of nanophthalmos will ultimately help us to provide potential markers for genetic diagnosis and development of innovative therapies for this condition. The goal of this review is to define nanophthalmos and provide a brief summary of the advances in the clinical characterization and genetic basis for nanophthalmos.

## 2. Methods

A Medline/PubMed search was performed using the terms “nanophthalmos,” “ocular development,” and “genetics” and their combinations. All studies published in English, Portuguese, or Spanish up to December 2017 were reviewed, and relevant publications were included in this review. The pertinent references of the selected articles were also included. All patient images were obtained with the permission of participating individuals or from parents of minor patients, as part of a study on nanophthalmos. This study was approved by the University of Michigan Institutional Review Board and complied with the US Health Insurance Portability and Accountability Act of 1996 and the Declaration of Helsinki.

## 3. Nanophthalmos: Definition and Clinical Features

Microphthalmos is a developmental disorder of the eye characterized by an axial length of at least 2 standard deviations below the mean for age [1]. This condition is classified as simple, when presented as an isolated finding, or complex, when accompanied by other malformations such as colobomas, anterior segment dysgenesis, lens abnormalities, and posterior segment anomalies [1]. It may also appear as a syndrome with other systemic features. These malformations result from a variety of genetic defects that induce abnormalities in early ocular embryogenesis [9–13].

**Figure 1:**
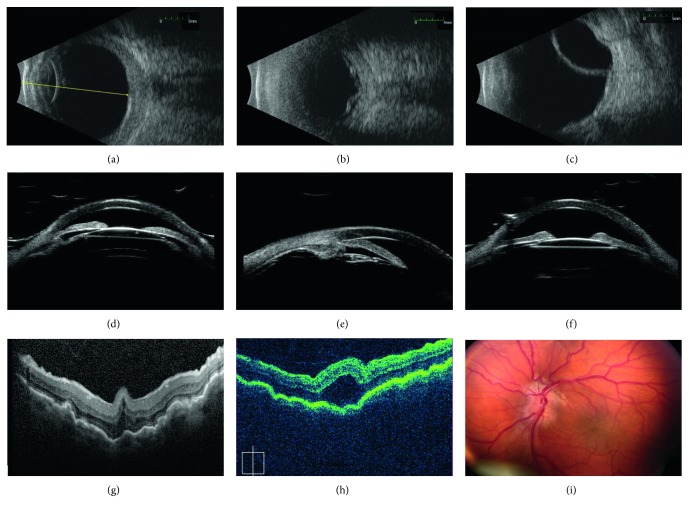
Typical ultrasonographic and retinal features of nanophthalmos. (a–c) B-scan ultrasounds showing features of nanophthalmos including short axial length, thickened sclera, and choroid (a), serous retinal detachment (b), and choroidal effusion (c). (d–f) Ultrasound biomicroscopy in a nanophthalmic eyes showing shallow anterior chamber (d), angle closure (e), and anterior rotation of the lens-iris diaphragm (f). (g) Heidelberg Spectralis OCT showing prominent choroidal and retinal folds in a small eye. (h) Zeiss Cirrus OCT showing foveoschisis and choroidal folds in a nanophthalmic eye. (i) Fundus photos in a patient with nanophthalmos and optic disc drusen, showing chorioretinal folds and crowded disc with mild vascular tortuousity.

Nanophthalmos is a special subtype of microphthalmia, in which the eye, although small, has preserved functionality and organization (Figure 1) [13, 14]. It usually presents as a small hyperopic eye set into a deep orbit, with narrow palpebral fissures [15, 16]. A high hypermetropic refractive error is an invariable feature, ranging from +8.00 D sphere to +25.00 or higher [2, 17]. However, the diagnostic criteria vary widely across the literature and considering only one parameter is simplistic. Wu et al. considered shallow anterior chamber, high hyperopia, axial length up to 21 mm, and posterior wall thickness greater than 1.7 mm as conditions to define nanophthalmic eyes [18]. Similarly, Yalvac et al. considered the same characteristics (with axial length defined as less than 20.5 mm) as diagnostic criteria but also added the high lens/eye volume ratio [19].

Another diagnostic issue that has been debated in the literature is the distinction between nanophthalmos and posterior microphthalmos. Posterior microphthalmos is described as a subtype of microphthalmia, in which the axial length is shortened in the posterior segment only. In this condition, the anterior segment of the eye has normal depth and angle configuration. Some investigators consider that nanophthalmos and posterior microphthalmos are synonymous [20]. The report that the reduction of the corneal diameter in high hyperopia is proportional to the axial shortening of the eye supports the hypothesis that these entities represent manifestations of the spectrum of hyperopia, rather than two completely different conditions. In addition, the fact that mutations in the same genes may cause both posterior microphthalmos and nanophthalmos reinforces this idea [20, 21].

However, other groups point to the clinical and structural differences between these conditions, such as the cornea size and curvature, anterior chamber depth, lens thickness, angle characteristics, and propensity for complications [2, 3, 20]. Relhan et al. [2] biometrically analyzed eyes of 38 patients with high hyperopia (defined in the study as greater than +7.00 D spherical equivalent on refraction), all of them with an axial length equal or less than 20.5 mm. In this study, they defined the patients with corneal diameters below 11.0 mm as nanophthalmic and those with corneal diameters greater than or equal to 11.0 mm as posterior microphthalmos. They found that nanophthalmic eyes have shallow anterior chamber depth, thicker lens, and steeper cornea, in comparison with posterior microphthalmic eyes [2]. They also reported different tendencies to complications: the incidence of angle-closure glaucoma was 69.23% in the nanophthalmos group versus 0% in the posterior microphthalmos group, while the incidence of macular folds was 0% versus 24%, respectively [2].

In addition to these clinical features, nanophthalmic eyes have abnormal collagen fibrils in each of the three layers of the sclera [22]. These abnormal fibers are thought to be the cause for the increase scleral thickness as mentioned above (Figure 1). In addition, the combination of increased scleral thickness and abnormal collagen also contributes to its inelasticity, which impairs vortex venous drainage and reduces transcleral flow of proteins [22]. These histopathologic features and anatomy described above are thought to be the mechanism by which nanophthalmic eyes develop complications of angle-closure glaucoma, uveal effusion syndrome, and retinal detachment [19, 22–28]. However, it is unclear whether the abnormal scleral structure is a primary or secondary effect of the genetic changes that induce nanophthalmos as many of the genes implicated in this condition are expressed in retina and retinal pigment epithelium [14, 29–32].

Other ocular findings include topographic corneal steepening and irregular astigmatism [33], absent or rudimentary foveal avascular zone [34], optic disc drusen, retinoschisis and foveoschisis and retinitis pigmentosa (RP) [35, 36], crowded optic disk, chorioretinal folds, and retinal cysts [37], central retinal vein occlusion [38], increased subfoveal choroidal thickness [39], and abnormalities in the retinal layers' thickness and distribution [40, 41] (Figure 1).

In summary, the described anatomical features and histopathology of the nanophthalmic eye explain the severe visual consequences in individuals with nanophthalmos. If the axial hyperopia is not corrected in early childhood, then this results in irreversible amblyopia. The unrecognized and untreated angle-closure glaucoma can lead to progressive optic nerve damage and blindness [26]. Furthermore, intraocular surgeries in nanophthalmic eyes have significant risks and complications, both intraoperatively and postoperatively [3, 25, 42, 43]. Proper preoperative planning and anatomic understanding can lead to good outcomes and improved quality of life in these patients [18], despite a nearly 40–60% rate of intraoperative complications [4, 22, 44, 45].

## 4. Genetic Aspects of Nanophthalmos

Nanophthalmos occurs due to arrested development of the eye in the early stages of embryogenesis. It is thought to have a strong genetic basis. There are many reported familial cases with autosomal-dominant and recessive forms of inheritance [26, 30, 46, 47]. However, nanophthalmos can also occur as a sporadic condition [23, 28, 48, 49], which may represent either environmental effects or somatic or new mutations that result in arrest of ocular growth.

To date, five genetic loci (Table 3) were reported to be linked to nanophthalmos: NNOS 2 is related to mutations in membrane frizzled-related protein (*MFRP*); NNOS 4 is related to mutations in *TMEM98*; MCOP6 is related to mutations in serine protease 56 (*PRSS56*) [50, 51]; and NNOS 1 and 3 were localized to chromosomal regions only (11p12-11q13 and 2q11-q14) [26, 29, 30]. Two additional genes, *CRB1* and *BEST1* (*VMD2*), have been implicated in nanophthalmos (Table 3) and have profound roles in photoreceptor and retinal pigment epithelial (RPE) function, respectively.

### 4.1. Membrane-Type Frizzled-Related Protein Gene (MFRP)

A significant number of cases of recessive nanophthalmos have been assigned to mutations in the membrane-type frizzled-related protein gene (*MFRP*, OMIM 606227) [29]. This gene is located in chromosome 11q23 and encodes a glycosylated transmembrane protein that has an extracellular frizzled-related cysteine-rich domain. Frizzled proteins are receptors involved in the regulation of growth, differentiation, and cell polarity during development through the Wnt signaling pathway [52, 53].

In humans, the *MFRP* gene is expressed in the retinal pigment epithelium and in the ciliary body [14, 29]. Outside of the eye, it can only be found at very low levels in the brain, likely accounting for the localized ocular phenotype in MFRP deficiency [29]. This gene seems to play an important role in both the ocular growth during childhood, functioning as a regulator of ocular size. It also has a role in maintenance of the RPE, which supports photoreceptor function [14, 54–56]. Mouse models of MFRP deficiency, such as rd6 (Mfrp^rd6^) and rdx (Mfrp^174delG^) mice, have flecked retina disorders and photoreceptor degeneration, supporting the importance of this gene for retinal and RPE physiology [57–60].

The link between eye size and RPE/ciliary body function has yet to be elucidated. It has been proposed that MFRP affects the physiologic mechanism of emmetropization, in which the refractive error is corrected by postnatal axial growth during the first six years of life [14]. Soundrarajan suggested that a complex regulatory network may influence the postnatal eye development and indicated the participation of another gene (*PRSS56*) in the same pathway [61]. Besides these, other proposed mechanisms for the role of the RPE/ciliary body in eye size include the mechanical stress effects and the inflammatory response observed in the retina [14, 62, 63]. Most recently, Velez et al. [64] found that introducing a normal copy of *Mfrp* gene through adenoviral-based gene therapy may reverse some of these pathogenic changes in Mfrp *rd6/rd6* mice. Specifically, subretinal injection of this vector resulted in rescue of photoreceptor death, normalization of retinal function, and regulation of eye length in adult mice. These findings suggest that gene therapy may be a viable option for this disease.

Mouse models of *Mfrp* loss-of-function have failed to demonstrate the full nanophthalmic phenotype observed in humans and instead present with predominant retinal degeneration [57–60]. This may be in part due to the differences in the lens size and ocular anatomy in mice and humans. Collery et al. proposed a new model using zebrafish (*Danio rerio*) that better mimics the human phenotype and may be useful in studying and better understanding this condition [62].

To date, several cases of MFRP mutations leading to reduced eye axial length have been reported. According to Wasmann et al. [65], by the time of the publication in 2014, there were 14 different described *MFRP* mutations: two of them were single amino acid substitutions at extremely conserved sites and 12 caused severe truncation of the protein. Since that time, three new mutations have been described [55], and additional known mutations have been reported in other populations [36, 66]. All of these cases presented with high hyperopia, but the effect of the mutation on retinal rod photoreceptor function was different between individuals, and the clinical spectrum of age of onset and severity of disease was quite variable [65]. The reason for this clinical variability may be a combination of the spectrum of genetic mutations in *MFRP* and other genetic or environmental modifiers that remain to be determined [67].

Wasmann et al. reported a case of two sisters with confirmed *MFRP* mutations. They both presented low visual acuity, high hyperopia, macular retinal folds, with the older sibling also having thickened sclera, and optic nerve head drusen [65]. The mutations in the *MFRP* gene have also been linked to the autosomal-recessive syndrome of posterior microphthalmos, retinitis pigmentosa, foveoschisis, and optic disc drusen [29, 35, 66–68]. These results represent the broad clinical spectrum of *MFRP* mutations, which occurs likely due to differences in early gene expression and environmental factors that shape the development of the eye.

### 4.2. Transmembrane Protein 98 Gene (TMEM98)

The transmembrane protein 98 (*TMEM98*, OMIM 615949) gene encodes a transmembrane protein that is universally expressed in the human body, including in the ocular tissues, such as iris, choroid, retinal pigment epithelium, and sclera. Its specific function still remains unclear, but it is hypothesized to lead to pathologic scleral pathologic thickening and secondary glaucoma development in nanophthalmic eyes or play a role in the development of the RPE [30, 31].

In a large pedigree, Awadalla and coworkers found a missense mutation in the *TMEM98* (A193P) that could be associated with autosomal-dominant nanophthalmos [30]. Although its pathogenic relationship with the disease was not clear, this association has been greatly strengthened by Khorram and colleagues' recent report of two novel *TMEM98* mutations (His196Pro and c.694_721delAG AATGAAGACTGGATCGAAGATGCCTCgtaagg) in autosomal-dominant nanophthalmic patients [31]. Additional studies are still needed to identify the specific role of this gene in the pathogenesis of nanophthalmos.

### 4.3. Protease Serine 56 (PRSS56)

*PRSS56*, also known as LOC646960, is located in the chromosome 2q37.1 and encodes a protein of 603 amino acids, which functions as a serine protease. It is suggested that it is expressed in the embryonic tissue, brain, testis, and eye [50]. There are reports of its association with nanophthalmos and posterior microphthalmos cases [21, 50, 69] although its physiologic and pathogenic mechanisms remain to be fully determined [21].

It has been reported that *PRSS56* is highly expressed in retinal ganglion cells of adult animals [50], and its presence in this tissue and in the brain cells suggest its relevance in the regulation of ocular development [69]. Nair et al. [51] demonstrated this role in the homozygous mutant mice Prss56^Grm4^, which showed shortened axial length and higher susceptibility to angle closure. Furthermore, they found that the differences in ocular size between mutant mice and wild-type controls were progressively greater after birth, with no significant difference prior to that time [51]. They found that the genetic background had a strong influence of magnitude of eye size differences between wild-type and mutant mice, suggesting the existence of genetic modifiers that influence eye growth in concert with *Prss56*. Soundararajan et al. also suggested that PRSS56 and MFRP may function through a common biological pathway that affects the emmetropization process, but nature of this interaction is still unclear [61].

### 4.4. Crumbs Homologue 1 Gene (CRB1)

Human *CRB1* is a 1406 amino acids transmembrane protein that localizes to photoreceptor inner segments and is vital for the neuronal development of the retina [70, 71]. The *CRB1* gene is located in chromosome 1, in the interval 1q31.2-1q32.1, and its mutations are classically associated with various heritable retinal dystrophies, including Leber Congenital Amaurosis [70, 72, 73]. Furthermore, some recent reports showed association of mutation in *CRB1* with nanophthalmos and retinitis pigmentosa [74, 75].

### 4.5. Bestrophin 1 (BEST1/VMD2)

The *BEST1 (VMD2)* gene is located on chromosome 11q12 and is primarily expressed in the RPE [32]. It encodes an integral membrane protein, bestrophin 1, localized predominantly in the basolateral plasma membrane of the RPE and most prominently near the macula [76, 77]. *BEST1* mutations are classically associated with Best vitelliform macular dystrophy (BVMD), a disease restricted to the macula. However, it has been reported to be in association with other widespread ocular abnormalities, such as autosomal-dominant vitreoretinochoroidopathy (ADVIRC) and autosomal-recessive bestrophinopathy (ARB), which are both associated with nanophthalmos [76]. Other studies also strongly suggest an association between *BEST1* mutations and angle-closure glaucoma [77, 78].

ADVIRC is a rare condition characterized by a peripheral circumferential hyperpigmented band with punctate white opacities in the retina, chorioretinal atrophy in the midperipheral or peripapillary retina, and vitreous fibrillary condensations [76, 79]. There are reports of association of this condition with nanophthalmos and a higher incidence of angle-closure glaucoma [79, 80].

ARB is also a rare condition characterized by macular and midperipheral subretinal whitish to yellowish deposits that may become scars and lead to decrease in visual acuity [81–83]. Patients are usually hyperopic and have a shallow anterior chamber and a higher propensity to angle-closure glaucoma [81–85].

### 4.6. Other Loci for Nanophthalmos

The autosomal-dominant nanophthalmos NNO1 (OMIM 600165) is caused by a defect on chromosome 11, between D11S905 and D11S987. This region may also be associated with severity of angle-closure glaucoma manifestations [26]. The precise genetic change at this locus has yet to be confirmed, though coding and regulatory mutations in BEST1 have been excluded as a cause (data not shown). Another form of autosomal-dominant disease, NNO3 (OMIM 611897), was described in a family with simple microphthalmia, microcornea, and high hyperopia, and it was reported to be linked to chromosome 2q11-14 [86].

## 5. Conclusion

With the progress of the imaging and surgical technologies, there have been significant advances in the diagnosis and management of the nanophthalmic eye. These have improved outcomes for individuals with such challenging eyes. Furthermore, substantial new discoveries in the genetics of nanophthalmos have led to the discovery of many new genes and pathways in the pathogenesis of this condition. These advances will ultimately improve early detection of this condition and provide novel avenues for treatment, including the possibility for gene therapy. Genetic diagnoses will facilitate genetic counseling for familial forms of this condition and may help to decrease amblyopia from uncorrected hyperopia, prevent vision loss from complications, and improve monitoring to minimize glaucoma and retinal complications from nanophthalmos.

## Figures and Tables

**Table 1:** The clinical spectrum of the small eye phenotype.

Anophthalmia	Absence of the eye
Simple microphthalmos	Short axial length due to global eye reduction with no other findings

Complex microphthalmos	Short axial length due to global eye reduction and associated ocular malformations (e.g., colobomas, persistent fetal vasculature, retinal dysplasia)

Relative anterior microphthalmos	Short axial length due to reduced anterior chamber dimension only, with normal posterior segment dimension and normal scleral thickness

Posterior microphthalmos	Short axial length due to reduced posterior segment dimension with normal anterior chamber dimensions

Nanophthalmos	Short axial length due to small anterior and posterior segments with thickened choroid and sclera and normal lens volume

**Table 2:** Clinical spectrum of eye size phenotypes based on axial length [5] and anterior segment features by anterior chamber depth and white-to-white corneal diameter [6–8].

	Axial length			
		Short (<21 mm)	Average (24 mm)	Long (>27 mm)
Anterior segment	Small (WTW < 11 mm; ACD < 3.0 mm)	Microphthalmos and nanophthalmos	Relative anterior microphthalmos	Complex dysgenesis
	Average (WTW∼11–12.5 mm; ACD∼3.3 mm)	Hyperopia posterior microphthalmos	Normal	Myopia
	Large (WTW > 12.5 mm ACD > 3.3 mm)	Complex dysgenesis	Megalocornea	Infantile or congenital glaucoma myopia

**Table 3:** Genes and phenotypes in nanophthalmos.

Gene (locus)	OMIM	Location	Inheritance	Gene expression (localization)	Gene function	Phenotypic characteristics of mutations
MFRP (NNO2)	606227	11q23.3	AR	RPE/CB (transmembrane)	Wnt signalling pathway effector	(i) Nanopthalmos, high hyperopia, and angle-closure glaucoma (ii) Retinitis pigmentosa, foveoschisis, and optic disc drusen syndrome

TMEM98 (NNO4)	615949	17q11.2	AD	RPE/CB/sclera (transmembrane)	Unknown	(i) High hyperopia, angle-closure glaucoma, and increased optic disc drusen

PRSS56 (MCOP6)	613858	2q37.1	AR	Retina/sclera (cytoplasmic)	Serine protease	(i) Nanophthalmos, angle-closure glaucoma, and high hyperopia (ii) Posterior microphthalmia

CRB1	604210	1q31.3	AR	Retina (transmembrane)	Controls cell polarity	(i) Nanophthalmos and retinitis pigmentosa (ii) Leber congenital amaurosis 8 (iii) Pigmented paravenous chorioretinal atrophy (iv) Retinitis pigmentosa

Best1/VMD2	607854	11q12	AD or AR	RPE/CB (transmembrane)	Chloride channel	(i) ADVIRC: autosomal-dominant vitreoretinochoroidopathy with nanophthalmos (ii) ARB: autosomal-recessive bestrophinopathy (iii) BVMD: best vitelliform macular dystrophy

Unknown (NNO3)	611897	2q11-q14	AD			(i) Microphthalmia, microcornea, and high hyperopia

Unknown (NNO1)	600165	11p12-11q13	AD			(i) High hyperopia, high lens/eye volume ratio, and angle-closure glaucoma

RPE: retinal pigment epithelium; CB: ciliary body.
